# Recovery of kidney function after dialysis initiation in children and adults in the US: A retrospective study of United States Renal Data System data

**DOI:** 10.1371/journal.pmed.1003546

**Published:** 2021-02-19

**Authors:** Elaine Ku, Raymond K. Hsu, Kirsten L. Johansen, Charles E. McCulloch, Mark Mitsnefes, Barbara A. Grimes, Kathleen D. Liu

**Affiliations:** 1 University of California San Francisco, Division of Nephrology, Department of Medicine, San Francisco, California, United States of America; 2 University of California San Francisco, Division of Pediatric Nephrology, Department of Pediatrics, San Francisco, California, United States of America; 3 University of California San Francisco, Department of Epidemiology and Biostatistics, San Francisco, California, United States of America; 4 Hennepin Healthcare and University of Minnesota, Department of Medicine, Division of Nephrology, Minneapolis, Minnesota, United States of America; 5 Cincinnati Children’s Hospital Medical Center, Department of Pediatrics, Division of Pediatric Nephrology and Hypertension, Cincinnati, Ohio, United States of America; 6 University of California San Francisco, Department of Anesthesia and Perioperative Care, San Francisco, California, United States of America; Royal Derby Hospital, UNITED KINGDOM

## Abstract

**Background:**

Little is known about factors associated with recovery of kidney function—and return to dialysis independence—or temporal trends in recovery after starting outpatient dialysis in the United States. Understanding the characteristics of individuals who may have the potential to recover kidney function may promote better recognition of such events. The goal of this study was to determine factors associated with recovery of kidney function in children compared with adults starting dialysis in the US.

**Methods and findings:**

We determined factors associated with recovery of kidney function—defined as survival and discontinuation of dialysis for ≥90-day period—in children versus adults who started maintenance dialysis between 1996 and 2015 according to the United States Renal Data System (USRDS) followed through 2016 in a retrospective cohort study. We also examined temporal trends in recovery rates over the last 2 decades in this cohort. Among 1,968,253 individuals included for study, the mean age was 62.6 ± 15.8 years, and 44% were female. Overall, 4% of adults (83,302/1,953,881) and 4% of children (547/14,372) starting dialysis in the outpatient setting recovered kidney function within 1 year. Among those who recovered, the median time to recovery was 73 days (interquartile range [IQR] 43–131) in adults and 100 days (IQR 56–189) in children. Accounting for the competing risk of death, children were less likely to recover kidney function compared with adults (sub-hazard ratio [sub-HR] 0.81; 95% CI 0.74–0.89, *p*-value <0.001; point estimates <1 indicating increased risk for a negative outcome). Non-Hispanic black (NHB) adults were less likely to recover compared with non-Hispanic white (NHW) adults, but these racial differences were not observed in children. Of note, a steady increase in the incidence of recovery of kidney function was noted initially in adults and children between 1996 and 2010, but this trend declined thereafter. The diagnoses associated with the highest recovery rates of recovery were acute tubular necrosis (ATN) and acute interstitial nephritis (AIN) in both adults and children, where 25%–40% of patients recovered kidney function depending on the calendar year of dialysis initiation. Limitations to our study include the potential for residual confounding to be present given the observational nature of our data.

**Conclusions:**

In this study, we observed that discontinuation of outpatient dialysis due to recovery occurred in 4% of patients with end-stage kidney disease (ESKD) and was more common among those with ATN or AIN as the cause of their kidney disease. While recovery rates rose initially, they declined starting in 2010. Additional studies are needed to understand how to best recognize and promote recovery in patients whose potential to discontinue dialysis is high in the outpatient setting.

## Abstract 2

In a retrospective study, Elaine Ku and colleagues investigate factors associated with recovery of kidney function among adults and children receiving dialysis reported in the United States Renal Data System.

## Author summary

**Why was this study done?:**

- Factors that predict a high potential for recovery of kidney function in dialysis patients are important to identify so that individuals who have the potential for recovery can be closely followed and their recovery recognized.
- The aim of this study was to determine the likelihood of recovery of kidney function following the start of maintenance dialysis in children and adults.

**What did the researchers do and find?:**

- We found that 4% of children and adults recovered kidney function after starting dialysis, but children were not more likely to recover kidney function compared with adults.
- Recovery rates improved initially over time in both children and adults but began to decline around 2010 and varied depending on age, race, and cause of kidney disease.

**What do these findings mean?:**

- Discontinuation of outpatient dialysis due to recovery of kidney function was observed in approximately 4% patients with end-stage kidney disease (ESKD) but was especially common among those with acute tubular necrosis (ATN) or acute interstitial nephritis (AIN) as the cause of their kidney disease.
- Additional studies are needed to understand how to best recognize and promote recovery in patients whose potential to discontinue dialysis is high in the outpatient setting.

## Introduction

Patients with acute kidney injury (AKI) in the setting of illnesses or hospitalization may often require dialysis initiation acutely [1–3]. A substantial proportion of these patients will survive their initial hospitalizations and be discharged to the outpatient setting with continued dialysis requirement [4–7]. However, subsets of these outpatients may have the potential to recover sufficient kidney function to eventually discontinue maintenance dialysis. Prior studies have reported that approximately 40% of patients who started dialysis after AKI and continued outpatient dialysis following hospital discharge had adequate recovery within 1 year [8]. Even in patients with progressive chronic kidney disease (CKD) who do not appear to suffer any acute insults prior to dialysis initiation, the decision to start dialysis may be based on factors that are not permanent (such as poor adherence to dietary restrictions or unreliable transportation to providers for close monitoring), so that even patients not thought to have an acute deterioration in kidney function may recover enough residual kidney function to maintain homeostasis and discontinue dialysis despite disease that was deemed consistent with end-stage kidney disease (ESKD) [9].

Given the growing incentives to shorten length of hospitalizations [10] and payer policy changes over the last decade surrounding AKI and ESKD care [11,12], temporal changes in renal recovery rates in the outpatient dialysis population have likely occurred [11,13]. In addition, the trend toward initiation of dialysis at progressively higher estimated glomerular filtration rate (eGFR; and potentially before dialysis is absolutely indicated) over the last 2 decades in the United States [14–17] may have led to a phenomenon in which subsets of patients who start dialysis subsequently “recover” from dialysis dependency; this phenomenon may also affect patients with dialysis-requiring AKI. The trend toward earlier dialysis initiation has been observed despite the publication of a large landmark trial in 2010 which demonstrated no survival benefit from earlier initiation of dialysis [14]. However, aside from single-center studies focused primarily on adult patients [3,18,19], the prevalence and predictors of recovery of kidney function after outpatient dialysis initiation for adults and children have not been well described on a national level.

The objective of this study was to determine the characteristics of adults (18 years or older) or children (<18 years) who recovered sufficient kidney function to discontinue dialysis for at least 90 days after starting outpatient maintenance dialysis treatment according to the United States Renal Data System (USRDS), the national ESKD registry that captures all patients undergoing maintenance dialysis in the US. We also examined temporal trends in the discontinuation of maintenance dialysis over time and compared these trends in adults and children. We chose to include both adults and children to enhance our understanding of how differences in disease etiology, practice patterns, and the competing risk of death (which is rare in children with ESKD) may be associated with the likelihood of discontinuing maintenance dialysis within 1 year of therapy initiation in outpatient facilities.

## Methods

### Study population and data source

We performed a retrospective study of all persons who started outpatient dialysis between January 1, 1996 and December 31, 2015 using data from the USRDS, the national ESKD registry. Demographic characteristics, the attributed cause of ESKD, insurance, zip code, date of ESKD onset, and race and ethnicity (categorized as non-Hispanic white [NHW], Hispanic, non-Hispanic black [NHB], Asian, or other) at incident ESKD were abstracted from the Centers for Medicare & Medicaid Services 2728 (CMS-2728) Medical Evidence Form (MEDEVID) and PATIENTS file in the USRDS. Zip code was used to determine median household income of patients’ neighborhoods using values from the American Community Survey between 2006 and 2010 [20]. Initial ESKD treatment modality (peritoneal dialysis versus hemodialysis) was determined at the first ESKD service date according to the RXHIST (treatment history) file. We examined the temporal relation between calendar year of dialysis initiation and recovery of kidney function using cubic splines.

### Definition of recovery and discontinuation of maintenance dialysis

Patients are registered within the USRDS after provider certification of their ESKD status via submission of the CMS-2728 MEDEVID file which is required within 45 days of dialysis initiation. The RXHIST file amalgamates data from Medicare Claims, CROWNWeb (a data management system through which Medicare-certified dialysis facilities report metrics to CMS), the CMS-2728 form, the CMS Death Notification Form, and the Organ Procurement Transplant Network Transplant files to update treatment status sequentially over time [21]. We used the RXHIST files to ascertain the initial ESKD treatment modality, date of chronic dialysis initiation, and changes in treatment modality including cessation of dialysis. Any patient who had a CMS-2728 form completed was eligible for inclusion for study.

Patients were considered to have recovered kidney function if they met all of the following: (1) they were noted to have recovered kidney function according to the RXHIST file and did not have an episode of restarting dialysis within 90 days of the date of recovery; (2) the patient did not die within 90 days of stopping dialysis treatment (and potentially withdraw from dialysis therapy); and (3) the patient did not receive a kidney transplant within 90 days of stopping dialysis. A 90-day dialysis-free period was required for recovery to remain consistent with definitions according to the USRDS (which also requires a 90-day period of dialysis independence) [21]. Patients who received a transplant at any time were censored, since even if recovery of kidney function occurred following kidney transplantation, it would be difficult to distinguish native kidney function from allograft function.

Follow-up began at the first episode of dialysis initiation according to the RXHIST file (and did not require a prespecified duration of dialysis for inclusion in our study) and ended at the date of renal recovery, death, 365 days following dialysis initiation, or administrative censoring date (December 31, 2016). We chose to focus on kidney recovery that occurred within 1 year of outpatient dialysis initiation as rates of recovery of kidney function thereafter were low. However, in sensitivity analyses, we also examined predictors of the outcome of recovery at any time during study follow-up.

### Predictors of recovery of kidney function

We first examined the characteristics of patients who recovered kidney function in children compared with adults. Characteristics of interest included age (as a 6-category variable), sex, race, insurance type, primary cause of ESKD, median neighborhood income, initial outpatient treatment modality (hemodialysis or peritoneal dialysis), calendar period (divided into 5-year intervals), region of the US (West, Northeast, South, and Midwest), and median neighborhood income.

Next, we used Fine and Gray models to examine the predictors of recovery of kidney function including candidate variables listed above in both univariable and multivariable analyses. Multivariable models included the covariates listed above and comorbid conditions including coronary artery disease, malignancy, heart failure, hypertension, diabetes, peripheral vascular disease, stroke, tobacco use, and drug use as reported on the CMS-2728 form. In these models, time was started from the first outpatient dialysis treatment session (and we did not require that patients have at least 90 days of dialysis to be included for study), death was treated as a competing event, and follow-up was censored at transplantation or administratively at 1 year after the first outpatient dialysis treatment. Of note, hazard ratios <1 in these models indicate a negative outcome (lower likelihood of recovery of kidney function) in this study. For these models, we included both adults and children in the same model so that comparisons could be made across the entire age spectrum. We also compared children (<18 years) and adults (18 years or older) as a 2-category predictor to determine the overall risk of recovery in univariable and multivariable analysis, adjusted for the same covariates as described above.

Next, we tested for differences in the associations between various characteristics and hazard of recovery in children versus adults by testing for interaction between sex, race, cause of kidney disease, initial treatment modality, insurance type, median income, calendar year of dialysis initiation, and region of the US with age at the time of dialysis initiation (<18 years versus ≥18 years) in unadjusted models. For factors where we found statistically significant interactions (*p* < 0.05), we then repeated our Fine and Gray analyses stratified by whether the patient was a child or adult at the time of dialysis initiation to understand how these factors differed among these populations in their association with recovery. We did not test for interactions between comorbidities given the low prevalence of comorbid conditions in the pediatric population.

### Subgroup analyses among diagnoses with high rates of recovery of kidney function

We identified the attributed causes of ESKD with the highest rates of recovery of kidney function for children and for adults. For each of these diagnoses, we determined the median time to recovery in children and adults separately among those who recovered kidney function and also the proportion who recovered at 30, 90, and 180 days of follow-up.

### Temporal trends in recovery of kidney function

To examine temporal trends in recovery of kidney function, we modeled time as a cubic spline with knots at 2000, 2004, 2008, and 2012 in linear regression models where the outcome was the average proportion of patients who recovered within 1 year of starting dialysis. We performed these analyses separately for children and adults and for patients with ESKD attributed to either acute tubular necrosis (ATN) or acute interstitial nephritis (AIN) which were the 2 diagnoses with the highest recovery rates in children and adults.

Next, we repeated our linear regression models with time as a cubic spline and recovery as the outcome, but stratified our models by whether the patient started dialysis early (≥10 mL/min/1.73 m^2^) versus late (<10 mL/min/1.73 m^2^) in adults and children separately. We determined the eGFR at dialysis initiation using the creatinine-based CKD-EPI equation in adults [22] and Schwartz equation [23] in children. Serum creatinine is routinely reported on the CMS-2728 form, which asks providers to certify the latest creatinine value within 45 days prior to the most recent episode of dialysis initiation. For eGFR determination, any serum creatinine reported more than 7 days after dialysis initiation was set as missing.

The University of California San Francisco Institutional Review Board considers this study not human subjects research and waived the requirement for ethical approval. Analyses were conducted in STATA 16 (Stata, Texas, US) and verified by a separate analyst using SAS 9.4 (SAS Institute, North Carolina, US) using USRDS data which authors had full access to and which is available to the public at https://www.usrds.org/for-researchers/simple-data-requests/. Due to extended run times for some analyses, we used a computationally more efficient method to perform the competing risks analyses. This consists of preprocessing the data, calculating time-dependent weights, and using standard Cox proportional hazards routines that incorporate the weights [24,25].

Our analytic plan was specified a priori during a meeting between coauthors with the goal of examining differences in recovery of kidney function within 1 year of starting dialysis in adults and children. Spline-based analyses to examine temporal trends in recovery of kidney function were added after completion of the main analyses given the recognition that these associations may have changed over time. Originally, the analytic plan was to only examine recovery of kidney function within 1 year of dialysis initiation, but at the request of reviewers during revision, we extended follow-up for recovery to the end of the study period and added this as an additional analysis. This study is reported as per the Strengthening the Reporting of Observational Studies in Epidemiology (STROBE) guidelines (S1 Checklist).

## Results

### Predictors of recovery of kidney function in adults and children

We identified 1,953,881 adults who started outpatient dialysis between 1996 and 2015. Overall, the mean age of the adult cohort was 63 ± 15.2 years, 44% were women, 28% were NHB, and 13% were Hispanic. Of these patients, 83,302 (4.3%) recovered sufficient kidney function and were able to gain independence from dialysis within a 1-year period, and an additional 12,360 (0.6%) individuals recovered sufficient kidney function to stop dialysis during longer term follow-up through the end of study. Among those recovering, the median time to recovery of kidney function in the adult cohort was 73 days (interquartile range [IQR] 43 to 132). Approximately 22% died (*N* = 439,552) prior to recovery of kidney function within a 1-year period.

We identified 14,372 children who started dialysis between 1996 and 2015. Overall, the mean age of the pediatric cohort was 10.3 ± 5.9 years, 45% were girls, 25% were NHB, and 27% were Hispanic. Of these patients, 547 (3.8%) recovered sufficient kidney function and gained independence from dialysis within a 1-year period. Approximately 4% of children died (*N* = 586) prior to recovery of kidney function within a 1-year period. The median time to recovery of kidney function in the pediatric cohort was 100 [IQR 56 to 189] days if recovery occurred.

Within 3 months of dialysis initiation, 5.8% of children (*N* = 831) and 0.5% of adults (*N* = 8,790) received kidney transplantation. Within 1 year of dialysis initiation, 30.3% of children (*N* = 4,350) and 2.4% of adults (*N* = 47,201) received kidney transplantation. Approximately 71% (*N* = 1,384,412) of adults and 62% of children (*N* = 8,889) were censored administratively as they did not reach any end point of interest by 1 year of follow-up. The proportion of children and adults who recovered within various timeframes are shown in S1 Fig.

Characteristics of adults and children who recovered kidney function are shown in Table 1. Qualitatively, a greater proportion of the adults who recovered were younger, more likely to be NHW, male, and more likely to have started outpatient therapy with hemodialysis (versus peritoneal dialysis) than those who did not recover kidney function. Qualitatively, a greater proportion of the children who recovered were also younger, but more likely to be of NHB or other race, and female (Table 1). Although starting hemodialysis as the initial treatment modality was also associated with more recovery than starting with peritoneal dialysis in children, a higher proportion of children starting outpatient peritoneal dialysis (3.5%, *N* = 243) recovered kidney function compared with adults (1.4%, *N* = 2,115, Table 1).

**Table 1:** Characteristics of patients who were included and who recovered kidney function after starting outpatient dialysis.

	Adults			Children		
Characteristic	Number of dialysis patients who recovered kidney function (*N*)	Total number of incident dialysis patients (*N*)	Proportion recovered (Row %)	Number of dialysis patients who recovered kidney function (*N*)	Total number of incident dialysis patients (*N*)	Proportion recovered (Row %)
Overall	83,302	1,953,881	4.3	547	14,372	3.8
Age category						
0–<5 years	–	–	–	175	3,310	5.3
5–<13 years	–	–	–	125	4,058	3.1
13–<18 years	–	–	–	247	7,004	3.5
18–<30 years	2,832	52,460	5.4	–	–	–
30–<65 years	40,790	918,902	4.4	–	–	–
65 years+	39,680	982,519	4	–	–	–
Male	48,689	1,085,926	4.5	239	7,859	3
Female	34,613	867,956	4	308	6,513	4.7
Race						
NHW	57,768	1,057,162	5.5	249	6,059	4.1
NHB	15,141	549,955	2.8	158	3,632	4.4
Hispanic	7,953	247,135	3.2	105	3,887	2.7
Asian	1,486	61,731	2.4	15	436	3.4
Other	954	37,898	2.5	20	358	5.6
Primary cause of kidney disease						
ATN	14,380	49,072	29.3	54	308	17.5
AIN	1,381	4,214	32.8	<11	14	–**
Glomerulonephritis	8,714	177,707	4.9	278	5,341	5.2
Diabetes	22,210	889,451	2.5	<11	80	–**
Hypertension	19,001	562,462	3.4	14	462	3
Urologic/cystic	1,746	68,183	2.6	20	1,292	1.6
Etiology unknown	4,723	67,132	7	22	1,030	2.1
Other/missing	11,147	135,660	8.2	153	5,845	2.6
Insurance						
None	423	11,257	3.8	<11	174	–
Medicaid/Medicare	40,040	984,387	4.1	245	6,933	3.5
Other/private	42,764	956,535	4.5	301	7,245	4.2
Missing	75	1,702	4.4	0	20	0
Calendar year of start						
1996–2000	10,817	401,502	2.7	116	3,491	3.3
2001–2005	18,216	478,509	3.8	152	3,886	3.9
2006–2010	28,280	525,682	5.4	168	3,697	4.5
2011–2015	25,989	548,188	4.7	111	3,298	3.4
Initial treatment modality						
Hemodialysis	81,187	1,801,543	4.5	304	7,326	4.2
Peritoneal dialysis	2,115	152,274	1.4	243	7,043	3.5

** Omitted when *N* is very low in accordance with USRDS publication policies.

AIN, acute interstitial nephritis; ATN, acute tubular necrosis; NHB, non-Hispanic black; NHW, non-Hispanic white; USRDS, United States Renal Data System.

### Subgroup analyses among diagnoses with high rates of recovery of kidney function

The attributed causes of kidney disease with the highest incidence of recovery among adults were AIN (33%, *N* = 1,381) and ATN (29%, *N* = 14,380, Table 1). In adults with these diagnoses, the median time to recovery among those who recovered were 49 [IQR 31 to 83] and 58 [IQR 36 to 98] days, respectively.

The attributed causes of kidney disease among children with the highest incidence of recovery were ATN (18%, *N* = 54), AIN (*N* <11) followed by glomerulonephritis (5%, *N* = 278, Table 1). The median time to recovery among those who recovered with these diagnoses were 136 days [IQR 48 to 229], 32 days [IQR 17 to 46], and 93 days [IQR 51 to 176], respectively.

### Predictors of recovery in adults versus children

Overall, after accounting for the competing risk of death, children were less likely to recover compared with adults (unadjusted analyses: sub-hazard ratio [sub-HR] 0.95; 95% CI 0.87 to 1.03; *p* = 0.24; adjusted analyses: sub-HR 0.81 [95% CI 0.74 to 0.89]; *p* < 0.001). Considering the full age spectrum, recovery was more likely to occur in the 0- to <5-year-old age groups (sub-HR 1.04; 95% CI 0.88 to 1.23, *p* = 0.64), although this did not reach statistical significance, and less likely in the 5- to <13-year-old (sub-HR 0.63; 95% CI 0.52 to 0.76, *p* < 0.001) and 13- to <18-year-old age groups (sub-HR 0.64; 95% CI 0.56 to 0.73, *p* < 0.001) in adjusted analyses (Table 2). Female sex (sub-HR 0.98; 95% CI 0.96 to 0.99, *p* < 0.001), NHB (sub-HR 0.52; 95% CI 0.51 to 0.53, *p* < 0.001), and Hispanic groups (sub-HR 0.66;95% CI 0.64 to 0.67; *p* < 0.001), those starting treatment with peritoneal dialysis (sub-HR 0.34; 95% CI 0.32 to 0.35, *p* < 0.001), those with urologic/cystic/congenital causes of kidney disease (sub-HR 0.08; 95% CI 0.08 to 0.09, *p* < 0.001) or diabetes (sub-HR 0.09; 95% CI 0.09 to 0.10, *p* < 0.001), and residence in the Northeastern part of the US (sub-HR 0.72; 95% CI 0.70 to 0.74, *p* < 0.001) were predictors associated with lower recovery rates in the overall population in multivariable models (Table 2). Similar findings were noted in sensitivity analyses when we extended follow-up to end of the study (S1 Table).

**Table 2:** Unadjusted and adjusted* Fine and Gray models for time to recovery from maintenance dialysis within 1 year of ESKD onset and effect modification by age (child [<18 years] versus adult [≥18 years]) at time of ESKD onset.

Sub-HR (95% CI)	Univariable model	*p-*value	Multivariable model	*p-*value
Age category (years)				
0–<5	0.99 (0.85–1.15)	0.86	1.04 (0.88–1.23)	0.64
5–<13	0.60 (0.50–0.71)	<0.001	0.63 (0.52–0.76)	<0.001
13–<18	0.68 (0.60–0.77)	<0.001	0.64 (0.56–0.73)	<0.001
18–30	Reference		Reference	
30–65	0.80 (0.77–0.83)	<0.001	0.95 (0.92–0.99)	0.02
65+	0.72 (0.70–0.75)	<0.001	0.75 (0.72–0.78)	<0.001
Female** (versus male)	0.89 (0.88–0.90)	<0.001	0.98 (0.96–0.99)	<0.001
Race**				
NHW	Reference		Reference	
NHB	0.50 (0.49–0.50)	<0.001	0.52 (0.51–0.53)	<0.001
Hispanic	0.58 (0.57–0.59)	<0.001	0.66 (0.64–0.67)	<0.001
Asian	0.43 (0.41–0.46)	<0.001	0.52 (0.49–0.55)	<0.001
Other	0.46 (0.43–0.49)	<0.001	0.54 (0.50–0.57)	<0.001
Primary cause of kidney disease**				
ATN	Reference		Reference	
AIN	1.18 (1.11–1.25)	<0.001	1.18 (1.11–1.25)	<0.001
Glomerulonephritis	0.14 (0.14–0.15)	<0.001	0.18 (0.17–0.18)	<0.001
Diabetes	0.07 (0.07–0.07)	<0.001	0.09 (0.09–0.10)	<0.001
Hypertension	0.10 (0.10–0.10)	<0.001	0.13 (0.13–0.13)	<0.001
Urologic/cystic/CAKUT	0.07 (0.07–0.08)	<0.001	0.08 (0.08–0.09)	<0.001
Etiology unknown	0.21 (0.20–0.21)	<0.001	0.24 (0.23–0.24)	<0.001
Other/missing	0.24 (0.23–0.25)	<0.001	0.26 (0.25–0.27)	<0.001
Peritoneal dialysis (versus hemodialysis)**	0.33 (0.31–0.34)	<0.001	0.34 (0.32–0.35)	<0.001
Calendar year of start**				
1996–2000	Reference		Reference	
2001–2005	1.42 (1.38–1.45)	<0.001	1.37 (1.34–1.40)	<0.001
2006–2010	2.02 (1.97–2.06)	<0.001	1.90 (1.86–1.94)	<0.001
2011–2015	1.77 (1.73–1.81)	<0.001	1.75 (1.71–1.79)	<0.001
Region of the US				
West	Reference		Reference	
Midwest	1.12 (1.09–1.14)	<0.001	0.96 (0.94–0.98)	0.001
South	1.06 (1.04–1.08)	<0.001	1.10 (1.08–1.12)	<0.001
Northeast	0.82 (0.80–0.84)	<0.001	0.72 (0.70–0.74)	<0.001
Median income (per US$)	1.00 (1.00–1.00)	<0.001	1.00 (1.00–1.00)	0.02
Insurance				
None	Reference		Reference	
Medicaid/Medicare	1.09 (0.99–1.20)	0.07	1.04 (0.94–1.14)	0.49
Private	1.21 (1.10–1.34)	<0.001	1.08 (0.98–1.19)	0.11

* Multivariable models are adjusted for age, sex, race, cause of kidney disease, dialysis modality, calendar year of dialysis initiation, region of the US, median neighborhood income, insurance, coronary artery disease, malignancy, heart failure, diabetes, hypertension, peripheral vascular disease, stroke, drug use, tobacco use, and account for the competing risk of death.

** Presence of an interaction (*p* < 0.05) between the factor of interest and age group (adult versus child).

We included *N* = 1,968,253 in univariable models and *N* = 1,933,687 in multivariable adjusted* models due to missing covariates.

AIN, acute interstitial nephritis; ATN, acute tubular necrosis; CAKUT, congenital anomalies of the kidney and urinary tract; ESKD, end-stage kidney disease; NHB, non-Hispanic black; NHW, non-Hispanic white; sub-HR, sub-hazard ratio.

However, some predictors of recovery differed in adults and children (presence of an interaction was detected) as shown in Table 3. Whereas women had a lower hazard of recovery than men (sub-HR 0.97; 95% CI 0.96 to 0.99, *p* < 0.001), girls had higher hazard of recovery than boys (sub-HR 1.47; 95% CI 1.22 to 1.76, *p* < 0.001; Table 2). Although NHB adults were less likely to recover kidney function than NHW adults (sub-HR 0.52; 95% CI 0.51 to 0.53, *p* < 0.001), there was no statistically significant difference between the recovery rates in NHB and NHW children (sub-HR 1.00; 95% CI 0.79 to 1.27, *p* = 0.98). Starting outpatient dialysis with peritoneal dialysis was associated with lower hazard of recovery compared with hemodialysis in both children and adults, but the effect size was more pronounced in adults (sub-HR 0.33; 95% CI 0.31 to 0.34, *p* < 0.001) compared with children (sub-HR 0.65; 95% CI 0.54 to 0.79, *p* < 0.001). Similar findings were noted when the outcome of recovery was followed until the end of the study (S2 Table).

**Table 3:** Unadjusted and adjusted* Fine and Gray models for time to recovery from maintenance dialysis within 1 year of ESKD onset with a focus on factors that differed between children and adults.

Sub-HR (95% CI)	Univariable model Adults	Multivariable model* Adults	*p-*value^$^	Univariable model Children	Multivariable model* Children	*p-*value^$^
Age category (years)						
0–<5	–	–	–	1.51 (1.24–1.83)	1.91 (1.50–2.44)	<0.001
5–<13	–	–	–	0.88 (0.70–1.08)	1.03 (0.82–1.30)	0.81
13–<18	–	–	–	Reference	Reference	
18–30	Reference	Reference			–	–
30–65	0.80 (0.77–0.83)	0.95 (0.91–0.99)	0.02		–	–
65+	0.72 (0.70–0.75)	0.74 (0.71–0.78)	<0.001		–	–
Female (versus male)	0.89 (0.87–0.90)	0.97 (0.96–0.99)	<0.001	1.57 (1.33–1.86)	1.47 (1.22–1.76)	<0.001
Race						
NHW	Reference	Reference		Reference	Reference	
Black	0.49 (0.48–0.50)	0.52 (0.51–0.53)	<0.001	1.06 (0.87–1.29)	1.00 (0.79–1.27)	0.98
Hispanic	0.58 (0.57–0.59)	0.66 (0.64–0.67)	<0.001	0.65 (0.52–0.82)	0.67 (0.52–0.86)	0.002
Asian	0.43 (0.41–0.46)	0.52 (0.49–0.55)	<0.001	0.83 (0.50–1.40)	0.72 (0.41–1.27)	0.26
Other	0.45 (0.42–0.48)	0.53 (0.50–0.57)	<0.001	1.37 (0.87–2.16)	1.43 (0.88–2.33)	0.15
Primary cause of kidney disease						
ATN	Reference	Reference		Reference	Reference	
Glomerulonephritis	0.14 (0.14–0.15)	0.18 (0.17–0.18)	<0.001	0.28 (0.21–0.37)	0.43 (0.30–0.61)	<0.001
AIN	1.18 (1.11–1.24)	1.18 (1.11–1.25)	<0.001	0.86 (0.20–3.79)	1.49 (0.31–7.19)	0.62
Diabetes	0.07 (0.07–0.07)	0.09 (0.09–0.10)	<0.001	0.27 (0.10–0.76)	0.28 (0.09–0.85)	0.03
Hypertension	0.10 (0.10–0.10)	0.13 (0.13–0.13)	<0.001	0.16 (0.09–0.29)	0.24 (0.13–0.47)	<0.001
Urologic/cystic/CAKUT	0.07 (0.07–0.08)	0.08 (0.08–0.09)	<0.001	0.08 (0.05–0.14)	0.11 (0.06–0.19)	<0.001
Etiology unknown	0.21 (0.20–0.21)	0.24 (0.23–0.25)	<0.001	0.11 (0.07–0.19)	0.16 (0.09–0.29)	<0.001
Other/missing	0.24 (0.24–0.25)	0.26 (0.25–0.27)	<0.001	0.14 (0.10–0.19)	0.19 (0.13–0.27)	<0.001
Peritoneal dialysis (versus hemodialysis)	0.30 (0.29–0.32)	0.33 (0.31–0.34)	<0.001	0.82 (0.70–0.98)	0.65 (0.54–0.79)	<0.001
Calendar year						
1996–2000	Reference	Reference		Reference	Reference	Reference
2001–2005	1.42 (1.39–1.45)	1.37 (1.34–1.40)	<0.001	1.18 (0.93–1.50)	1.37 (1.05–1.78)	0.02
2006–2010	2.02 (1.98–2.07)	1.90 (1.86–1.95)	<0.001	1.37 (1.08–1.73)	1.49 (1.15–1.92)	0.002
2011–2015	1.77 (1.73–1.81)	1.75 (1.71–1.79)	<0.001	1.01 (0.78–1.31)	1.05 (0.80–1.38)	0.73

* Multivariable models are adjusted for age, sex, race, cause of kidney disease, dialysis modality, calendar year of dialysis initiation, region of the US, median neighborhood income, insurance, coronary artery disease, malignancy, heart failure, diabetes, hypertension, peripheral vascular disease, stroke, drug use, tobacco use, and account for the competing risk of death.

$ *p-*values shown are for multivariable models.

We included *N* = 1,953,881 adults and 14,372 children in univariable analyses. We included *N* = 1,920,951 adults and *N* = 12,736 children in multivariable adjusted* analyses.

AIN, acute interstitial nephritis; ATN, acute tubular necrosis; CAKUT, congenital anomalies of the kidney and urinary tract; ESKD, end-stage kidney disease; NHW, non-Hispanic white; sub-HR, sub-hazard ratio.

### Temporal trends in recovery of kidney function in adults versus children

**Fig 1:**
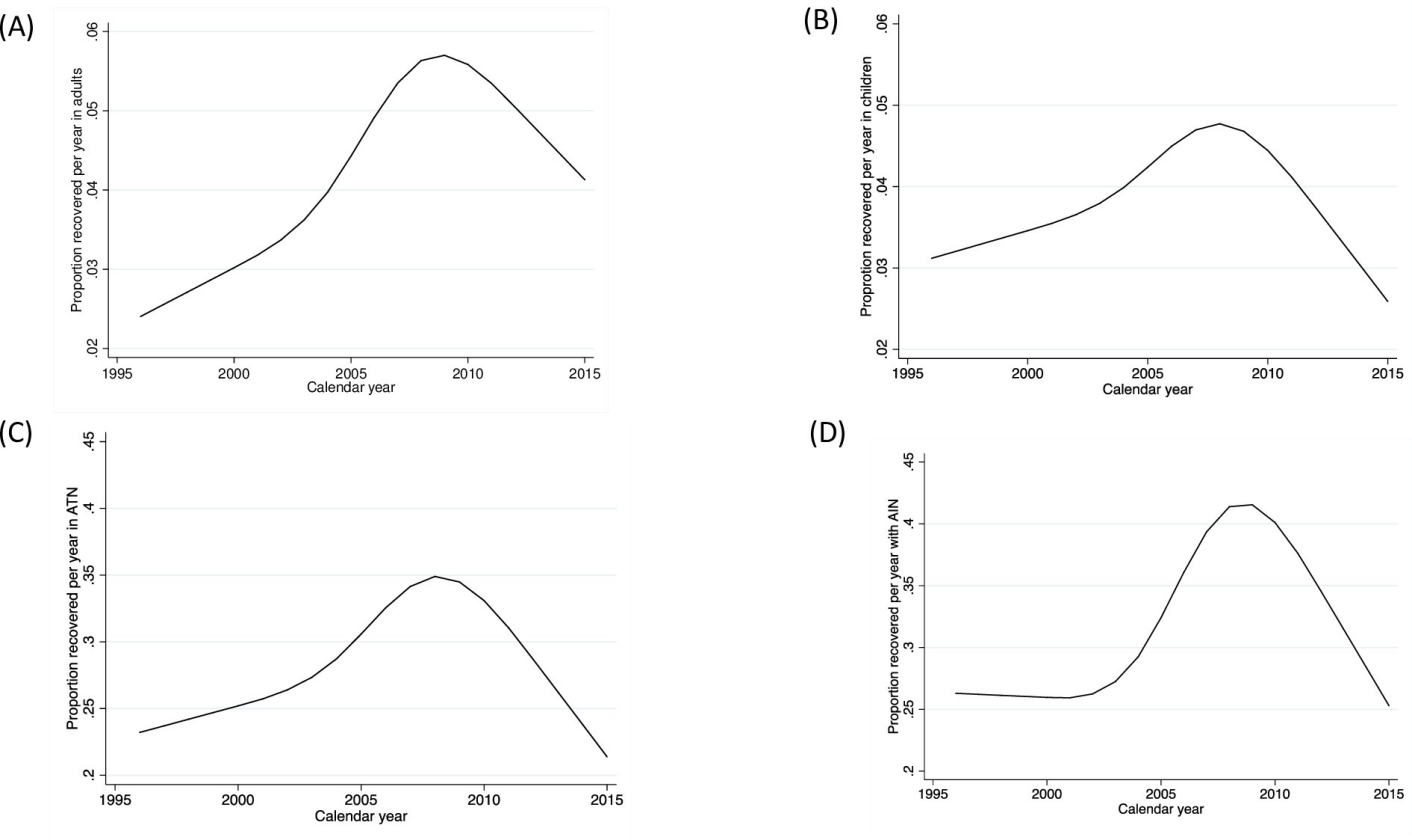
Temporal trends in recovery of kidney function within 1 year of dialysis. AIN, acute interstitial nephritis; ATN, acute tubular necrosis.

When we examined temporal trends in the recovery of kidney function among adults, recovery of kidney function was noted to peak qualitatively in 2010 and began to decline in the last 5-year calendar period (Fig 1A). In children, similar trends were noted qualitatively, but the magnitude of the improvement in recovery rates prior to 2010 was qualitatively less pronounced than that observed in adults (Fig 1B).

When we examined temporal trends in recovery of kidney function among adult and pediatric patients with a diagnosis of ATN (Fig 1C), trends were qualitatively similar to that observed in the overall cohort (Fig 1A and 1B), but at the peak rate of recovery around 2009, 36% (*N* = 1,370 out of 3,786) of patients were recovering kidney function within 1 year of starting dialysis. In AIN, rates of recovery exceeded 40% (*N* = 135 out of 312) around 2010 and then began to decline thereafter as seen qualitatively in Fig 1D.

Trends in recovery of kidney function were statistically significantly different in adults (sub-HR 1.90; 95% CI 1.86 to 1.95, *p* < 0.001) versus children (sub-HR 1.49; 95% CI 1.15 to 1.92, *p* = 0.002) in the 2006 to 2010 period (Table 3) as well as in the 2011 to 2015 period (sub-HR 1.75; 95% CI 1.71 to 1.79, *p* < 0.001 in adults versus sub-HR 1.05; 95% CI 0.80 to 1.38, *p* = 0.73 in children). In particular, children in the most recent 5-year period (2011 to 2015) did not have a statistically significantly higher hazard of recovery of kidney function (sub-HR 1.05; 95% CI 0.80 to 1.38; *p* = 0.73) compared with children starting dialysis between 1996 and 2000 (the reference comparator).

### Relation between temporal trends in recovery and timing of dialysis initiation at higher versus lower eGFR

**Fig 2:**
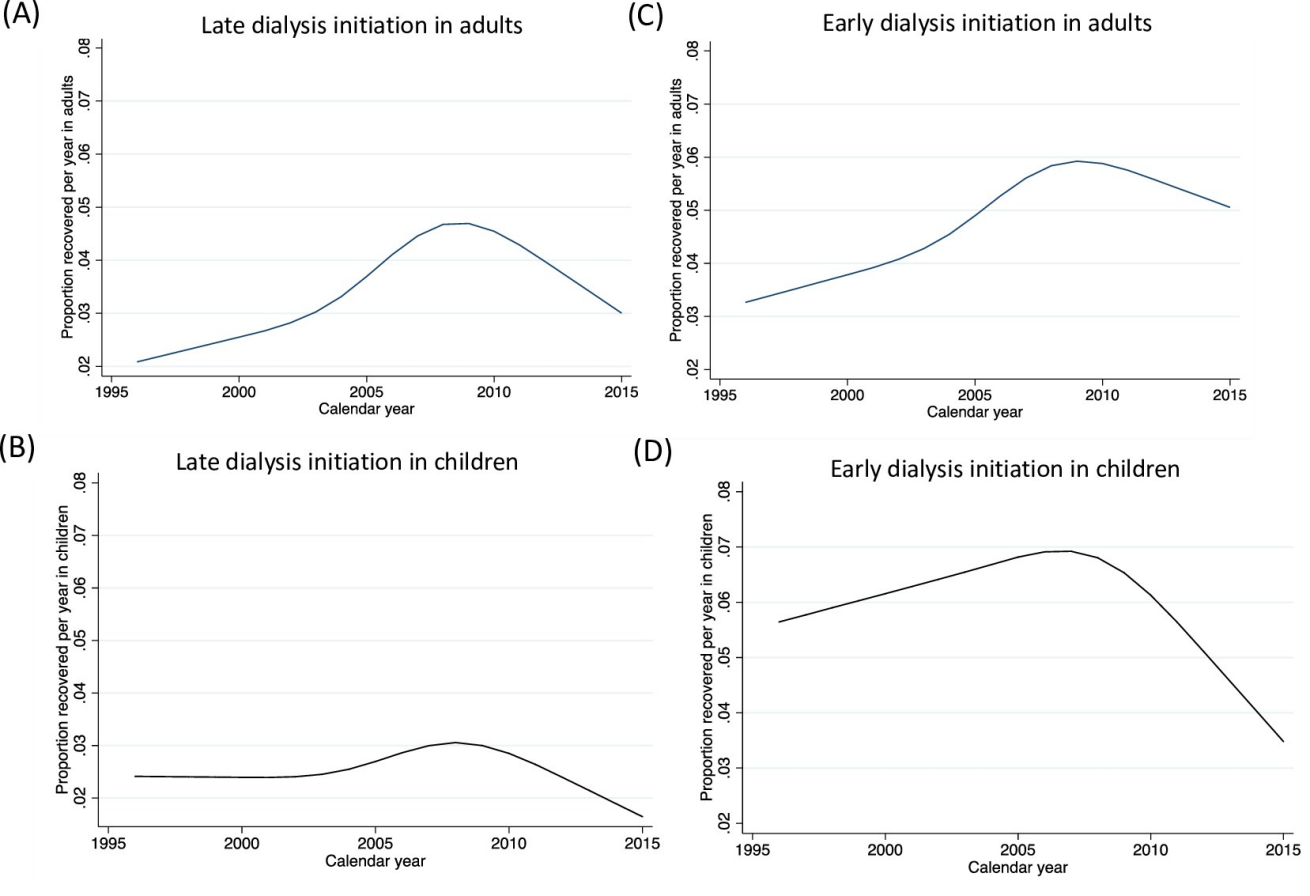
Temporal trends in recovery in adults versus children stratified by early versus late dialysis initiation. eGFR, estimated glomerular filtration rate.

We further explored qualitatively whether the temporal trends in recovery of kidney function would differ among those who were started on dialysis at higher versus lower eGFR. As shown in Fig 2A and 2B, recovery was qualitatively lower in those who had an eGFR <10 mL/min/1.73 m^2^ at dialysis initiation compared with those with earlier dialysis initiation (eGFR ≥10 mL/min/1.73 m^2^, Fig 2C and 2D). Recovery rates improved initially for adults regardless of whether they underwent early or late initiation and subsequently declined over time as seen qualitatively in Fig 2A and 2C. In contrast, recovery rates were stably low for children who started dialysis late and did not improve as substantially over time compared to children who started dialysis early as seen qualitatively in Fig 2B and 2D.

## Discussion

Many patients who start outpatient dialysis therapy inquire about their chances of discontinuing dialysis over time. However, few studies have focused on understanding the incidence, predictors, and temporal trends of recovery among patients receiving outpatient dialysis treatment. In this study, we examined the incidence of recovery of kidney function among persons starting outpatient dialysis treatment over the last 2 decades. We found that overall, 4% of the population who started outpatient dialysis recovered sufficient kidney function to discontinue maintenance dialysis within 1 year. Recovery rates ranged between 10% and 15% within the first 30 days of dialysis initiation, but nearly half of patients who recovered kidney function did so within 90 days after dialysis initiation. Few patients recovered after 180 days of outpatient chronic dialysis. Adults were more likely to recover and become dialysis independent compared with children, but in both populations, ATN and AIN were associated with the highest recovery rates, and these recovery rates were as high as 25% to 45% depending on the calendar year of study. However, rates of recovery differed by etiology of kidney disease.

Although we had hypothesized that children would have better potential for recovery of kidney function given their lower prevalence of age-related comorbidities and lower likelihood of long-standing CKD, we found lower rates of recovery in the outpatient setting among children compared with adults. Differences in practice patterns among adult and pediatric practitioners may explain the observed differences in the risk of recovery in the adult versus pediatric population. In pediatrics, there may be a greater tendency to monitor patients with dialysis-requiring AKI in the inpatient setting for recovery (rather than discharge these patients to outpatient dialysis units), as it can be logistically challenging to identify outpatient dialysis units that are willing to treat younger children (outside of large academic centers) [26]. There may also be less pressure to discharge children from inpatient to outpatient care, especially given the complexities of ESKD care in the outpatient setting and training required for pediatric caregivers. In addition, a larger proportion of children with ESKD in our cohort were treated with peritoneal dialysis, which was a risk factor for non-recovery in both the pediatric and adult population. Because peritoneal dialysis catheters may be more difficult to place and remove than tunneled hemodialysis catheters, providers may be more conservative about using peritoneal dialysis as a treatment modality unless patients have strong evidence of end-stage disease. We acknowledge, however, that some pediatric centers do offer peritoneal dialysis as an acute treatment modality in the setting of AKI (which is less common in adult practice in the US) [27].

We also noted differences in recovery by race in the adult and pediatric populations. In adults, NHB individuals had a lower probability of recovery compared with NHW individuals, but this racial disparity was not observed in children. It is likely that socioeconomic factors such as access to insurance and routine healthcare prior to ESKD may contribute to these observations, as children typically have universal healthcare coverage and better access to care [28, 29]. The steady decrease in recovery of kidney function after 2012 is consistent with policy clarifications that may have further disincentivized the acceptance of patients with AKI for dialysis treatment at outpatient facilities between 2012 and 2017. However, the decline in recovery rates began even prior to the onset of this policy change in 2012, the reasons for which are unclear.

A steady increase in the number of patients who are receiving dialysis after AKI in the US population [30] has been noted over time [21]. Given improvements in the survival of patients with AKI-requiring dialysis to hospital discharge [31], it is possible that this contributed to the better rates of recovery of kidney function up until 2010 [11]. However, this trend was noteworthy among those starting dialysis with an eGFR above 10 mL/min/1.73 m^2^ in both children and adults. The frequently unpredictable nature of CKD progression may lead some practitioners to conservatively start dialysis early, and a smaller degree of recovery in these patients may be necessary in order to be able to discontinue dialysis. We do acknowledge that policy clarifications which emphasized that Medicare patients receiving treatment at outpatient dialysis facilities in the US for AKI would not be reimbursed for their outpatient treatments were released in 2012 [11,12]. This clarification to Medicare payment policies may have delayed hospital discharge of inpatients who otherwise were ready for outpatient care while awaiting recovery of kidney function and could potentially contribute to a decrease in the rate of recovery of kidney function in outpatient facilities thereafter.

Our study has practice and policy implications. First, nephrology providers may need to consider earlier and more frequent in-person visits at dialysis units, more vigilant assessments of residual kidney function, and changes in practice patterns (such as avoidance of excessive ultrafiltration and intradialytic hypotension) for the subset who may recover kidney function [32–34]. In addition, the frequent pressure to make the determination of whether an individual has ESKD may lead to misclassification of patients and inappropriate resource utilization, such as transportation and insurance benefits that may come with the diagnosis of ESKD. Whether more recent policy changes which now allow for the reimbursement of dialysis provisions for AKI in outpatient facilities have influenced temporal and practice pattern changes remains to be determined [34].

The strengths of our study include the large size of the national cohort, the contemporary nature of the data, and the inclusion of a racially and ethnically diverse group of adults and children. Our follow-up of participants is also longer than most prior studies which have been primarily single-center studies and may have limited data on the long-term outcomes of patients after the start of dialysis in the outpatient setting. Limitations include potential errors in data and missing data from the CMS-2728 forms that may have led to potential misclassification of predictors of recovery and lack of data surrounding whether nephrotoxic agents were used following AKI which may have delayed recovery of kidney function. We lack substantial data surrounding care of patients prior to dialysis initiation, and we are unable to clearly delineate whether patients had baseline CKD and subsequently developed AKI or the exact nature of the trajectory of kidney function prior to the initiation of dialysis. We believe our study population includes patients with more severe AKI who continued to require dialysis in the outpatient setting, and thus our results do not generalize to patients who may have developed AKI and recovered kidney function prior to hospital discharge or to patients in other countries where dialysis care may differ from that in the US. We also acknowledge that changes to care of patients with AKI may have occurred with most recent policy changes surrounding reimbursement for the dialysis of patients with AKI, which may limit the applicability of our findings following such policy changes. Finally, given the observational nature of our data, residual confounding may be present.

In conclusion, we note that recovery of adequate kidney function for discontinuation of outpatient dialysis occurs in 4% of patients who were noted to have ESKD. Close monitoring of patients within the first 6 months of dialysis initiation may be prudent, especially among those with ATN, AIN, or in children, glomerulonephritis. Further studies are needed to understand factors that may improve the chances of recovery of kidney function in the outpatient setting, and strategies are needed to maximize the potential for recovery.

## Supporting information

S1 STROBE Checklist STROBE checklist for the manuscript. STROBE, Strengthening the Reporting of Observational Studies in Epidemiology.
(DOC)

S1 Table Adjusted* Fine and Gray models for time to recovery from maintenance dialysis at any point during follow-up after ESKD onset. ESKD, end-stage kidney disease.
(DOCX)

S2 Table Adjusted* Fine and Gray models for time to recovery from maintenance dialysis at any point during follow-up after ESKD onset with a focus on factors that differed between children and adults. ESKD, end-stage kidney disease.
(DOCX)

S1 Fig Percent of children and adults recovering kidney function within different time intervals of interest. (TIF)

## Abbreviations

AIN: acute interstitial nephritis
AKI: acute kidney injury
ATN: acute tubular necrosis
CMS: Centers for Medicare & Medicaid Services
eGFR: estimated glomerular filtration rate
ESKD: end-stage kidney disease
IQR: interquartile range
MEDEVID: Medical Evidence Form
NHB: non-Hispanic black
NHW: non-Hispanic white
STROBE: Strengthening the Reporting of Observational Studies in Epidemiology
sub-HR: sub-hazard ratio
USRDS: United States Renal Data System

## References

[1] Gautam SC, Brooks CH, Balogun RA, Xin W, Ma JZ, Abdel-Rahman EM. Predictors and Outcomes of Post-Hospitalization Dialysis Dependent Acute Kidney Injury. Nephron. 2015;131(3):185–90. 10.1159/000441607 26524288

[2] Kellum JA, Lameire N. Diagnosis, evaluation, and management of acute kidney injury: a KDIGO summary (Part 1). Crit Care. 2013;17(1):204. 10.1186/cc11454 23394211 PMC4057151

[3] Pajewski R, Gipson P, Heung M. Predictors of post-hospitalization recovery of renal function among patients with acute kidney injury requiring dialysis. Hemodial Int. 2018;22(1):66–73. 10.1111/hdi.12545 28296033

[4] Chu JK, Folkert VW. Renal function recovery in chronic dialysis patients. Semin Dial. 2010;23(6):606–13. 10.1111/j.1525-139X.2010.00769.x 21166875

[5] Rottembourg J, Issad B, Allouache M, Jacobs C. Recovery of renal function in patients treated by CAPD. Adv Perit Dial. 1989;5:63–6. 2577429

[6] Schiffl H. Renal recovery from acute tubular necrosis requiring renal replacement therapy: a prospective study in critically ill patients. Nephrol Dial Transplant. 2006;21(5):1248–52. 10.1093/ndt/gfk069 16449291

[7] Macdonald JA, McDonald SP, Hawley CM, Rosman J, Brown F, Wiggins KJ, et al. Recovery of renal function in end-stage renal failure—comparison between peritoneal dialysis and haemodialysis. Nephrol Dial Transplant. 2009;24(9):2825–31. 10.1093/ndt/gfp216 19443649

[8] Pajewski R, Gipson P, Heung M. Predictors of post-hospitalization recovery of renal function among patients with acute kidney injury requiring dialysis. Hemodial Int. 2017. 10.1111/hdi.12545 28296033

[9] Council on Ethical and Judicial Affairs. Black-white disparities in health care. JAMA. 1990;263(17):2344–6. 10.1001/jama.1990.03440170066038 2182918

[10] Kidney Disease Improving Global Outcomes Writing Group. Chapter 5: Referral to specialists and models of care. Volume 3, Issue 1. 2013 [cited 2019 May 16]. p.112-119. Available from: http://www.sciencedirect.com/science/article/pii/S2157171615311059. 10.1038/kisup.2012.68 25599001 PMC4284442

[11] Heung M. Outpatient Dialysis for Acute Kidney Injury: Progress and Pitfalls. Am J Kidney Dis. 2019. 10.1053/j.ajkd.2019.03.431 31204193

[12] Centers for Medicare & Medicaid Services (CMS), HHS. Medicare program; revisions to payment policies under the physician fee schedule for calendar year 2004. Final rule with comment period. Fed Regist. 2003;68:63195. 14610760

[13] Wright JT Jr, Williamson JD, Whelton PK, Snyder JK, Sink KM, Rocco MV, et al. A Randomized Trial of Intensive versus Standard Blood-Pressure Control. N Engl J Med. 2015;373(22):2103–16. 10.1056/NEJMoa1511939 26551272 PMC4689591

[14] Cooper BA, Branley P, Bulfone L, Collins JF, Craig JC, Fraenkel MB, et al. A randomized, controlled trial of early versus late initiation of dialysis. N Engl J Med. 2010;363(7):609–19. 10.1056/NEJMoa1000552 20581422

[15] Ferguson TW, Garg AX, Sood MM, Rigatto C, Chau E, Komenda P, et al. Association Between the Publication of the Initiating Dialysis Early and Late Trial and the Timing of Dialysis Initiation in Canada. JAMA Intern Med. 2019. 10.1001/jamainternmed.2019.0489 31135821 PMC6547160

[16] Rosansky SJ, Clark WF, Eggers P, Glassock RJ. Initiation of dialysis at higher GFRs: is the apparent rising tide of early dialysis harmful or helpful? Kidney Int. 2009;76(3):257–61. 10.1038/ki.2009.161 19455195

[17] Susantitaphong P, Altamimi S, Ashkar M, Balk EM, Stel VS, Wright S, et al. GFR at initiation of dialysis and mortality in CKD: a meta-analysis. Am J Kidney Dis. 2012;59(6):829–40. 10.1053/j.ajkd.2012.01.015 22465328 PMC3395227

[18] Hickson LJ, Chaudhary S, Williams AW, Dillon JJ, Norby SM, Gregoire JR, et al. Predictors of outpatient kidney function recovery among patients who initiate hemodialysis in the hospital. Am J Kidney Dis. 2015;65(4):592–602. 10.1053/j.ajkd.2014.10.015 25500361 PMC4630340

[19] Hickson LJ, Chaudhary S, Williams AW, Dillon JJ, Norby SM, Gregoire JR, et al. Predictors of Outpatient Kidney Function Recovery Among Patients Who Initiate Hemodialysis in the Hospital. Am J Kidney Dis. 2014. 10.1053/j.ajkd.2014.10.015 25500361 PMC4630340

[20] Population Studies Center. Zip Code Characteristics: Mean and Median Household Income University of Michigan: Institute for Social Research. 2010 [cited 2017 Feb 10]. Available from: http://www.psc.isr.umich.edu/dis/census/Features/tract2zip/index.html.

[21] United States Renal Data System. 2018 USRDS annual data report: Epidemiology of kidney disease in the United States. National Institutes of Health NIDDK.

[22] Inker LA, Schmid CH, Tighiouart H, Eckfeldt JH, Feldman HI, Greene T, et al. Estimating glomerular filtration rate from serum creatinine and cystatin C. N Engl J Med. 2012;367(1):20–9. 10.1056/NEJMoa1114248 22762315 PMC4398023

[23] Schwartz GJ, Muñoz A, Schneider MF, Mak RH, Kaskel F, Warady BA, et al. New equations to estimate GFR in children with CKD. J Am Soc Nephrol. 2009;20(3):629–37. 10.1681/ASN.2008030287 19158356 PMC2653687

[24] Kidney Disease: Improving Global Outcomes (KDIGO) CKD Work Group. KDIGO 2012 Clinical Practice Guideline for the Evaluation and Management of Chronic Kidney Disease. / Levin, Adeera; Stevens, Paul E.; Bilous, Rudy W.; Coresh, Josef; De Francisco, Angel LM.; De Jong, Paul E.; Griffith, Kathryn E.; Hemmelgarn, Brenda R.; Iseki, Kunitoshi; Lamb, Edmund J.; Levey, Andrew S.; Riella, Miguel C.; Shlipak, Michael G.; Wang, Haiyan; White, Colin T.; Winearls, Christopher G. [cited 2019 Jun 1]. Available from: https://kdigo.org/guidelines/ckd-evaluation-and-management/.

[25] Geskus RB. Cause-specific cumulative incidence estimation and the fine and gray model under both left truncation and right censoring. Biometrics. 2011;67(1):39–49. 10.1111/j.1541-0420.2010.01420.x 20377575

[26] Chand DH, Swartz S, Tuchman S, Valentini RP, Somers MJ. Dialysis in Children and Adolescents: The Pediatric Nephrology Perspective. Am J Kidney Dis. 2017;69(2):278–86. 10.1053/j.ajkd.2016.09.023 27940060

[27] Guzzo I, de Galasso L, Mir S, Bulut IK, Jankauskiene A, Burokiene V, et al. Acute dialysis in children: results of a European survey. J Nephrol. 2019;32(3):445–51. 10.1007/s40620-019-00606-1 30949986

[28] Kucirka LM, Grams ME, Lessler J, Hall EC, James N, Massie AB, et al. Association of race and age with survival among patients undergoing dialysis. JAMA. 2011;306(6):620–6. 10.1001/jama.2011.1127 21828325 PMC3938098

[29] Rhee CM, Lertdumrongluk P, Streja E, Park J, Moradi H, Lau WL, et al. Impact of age, race and ethnicity on dialysis patient survival and kidney transplantation disparities. Am J Nephrol. 2014;39(3):183–94. 10.1159/000358497 24556752 PMC4024458

[30] Hsu RK, McCulloch CE, Dudley RA, Lo LJ, Hsu CY. Temporal Changes in Incidence of Dialysis-Requiring AKI. J Am Soc Nephrol. 2013;24(1):37–42. 10.1681/ASN.2012080800 23222124 PMC3537221

[31] Brown JR, Rezaee ME, Hisey WM, Cox KC, Matheny ME, Sarnak MJ. Reduced Mortality Associated with Acute Kidney Injury Requiring Dialysis in the United States. Am J Nephrol. 2016;43(4):261–70. 10.1159/000445846 27161485 PMC4899228

[32] Heung M, Faubel S, Watnick S, Cruz DN, Koyner JL, Mour G, et al. Outpatient Dialysis for Patients with AKI: A Policy Approach to Improving Care. Clin J Am Soc Nephrol. 2015;10(10):1868–74. 10.2215/CJN.02290215 26220818 PMC4594066

[33] Harel Z, Wald R, Bargman JM, Mamdani M, Etchells E, Garg AX, et al. Nephrologist follow-up improves all-cause mortality of severe acute kidney injury survivors. Kidney Int. 2013. 10.1038/ki.2012.451 23325077

[34] Cerdá J, Liu KD, Cruz DN, Jaber BL, Koyner JL, Heung M, et al. Promoting Kidney Function Recovery in Patients with AKI Requiring RRT. Clin J Am Soc Nephrol. 2015. 10.2215/CJN.01170215 26138260 PMC4594060

