# Supplementary material for: Recovery of kidney function after dialysis initiation in children and adults in the US: A retrospective study of United States Renal Data System data
Source: PLoS Med. 2021 Feb 19;18(2):e1003546. doi: 10.1371/journal.pmed.1003546 (PMC7935284; doi:10.1371/journal.pmed.1003546)
Supplement: S1 STROBE Checklist — STROBE, Strengthening the Reporting of Observational Studies in Epidemiology. (DOC) [file pmed.1003546.s001.doc]

STROBE Statement—checklist of items that should be included in reports of observational studies

|  | Item No | Recommendation |
| --- | --- | --- |
| **Title and abstract** | 1 | (*a*) Indicate the study’s design with a commonly used term in the title or the abstract. **This is in the title.** |
| (*b*) Provide in the abstract an informative and balanced summary of what was done and what was found. **This can be found in the abstract.** |
| Introduction | | |
| Background/rationale | 2 | Explain the scientific background and rationale for the investigation being reported **This is in the first two paragraphs of the introduction.** |
| Objectives | 3 | State specific objectives, including any prespecified hypotheses. **This is in the third paragraph of the introduction.** |
| Methods | | |
| Study design | 4 | Present key elements of study design early in the paper. **This is in the first paragraph of the methods.** |
| Setting | 5 | Describe the setting, locations, and relevant dates, including periods of recruitment, exposure, follow-up, and data collection **This is in the first paragraph of the methods.** |
| Participants | 6 | (*a*) *Cohort study*—Give the eligibility criteria, and the sources and methods of selection of participants. Describe methods of follow-up. **This is in the first and second paragraph of the methods.** |
| (*b*) *Cohort study*—For matched studies, give matching criteria and number of exposed and unexposed – **Not applicable** |
| Variables | 7 | Clearly define all outcomes, exposures, predictors, potential confounders, and effect modifiers. Give diagnostic criteria, if applicable  **This is in the section on Predictors of kidney function recovery and Definition of Recovery and discontinuation of maintenance dialysis.** |
| Data sources/ measurement | 8* | For each variable of interest, give sources of data and details of methods of assessment (measurement). Describe comparability of assessment methods if there is more than one group **This is in the Methods section – section on predictors of kidney function recovery.** |
| Bias | 9 | Describe any efforts to address potential sources of bias ✔ **Examination of transplant/death rates as competing risks described in the second paragraph under the section Predictors of Kidney Function Recovery in the Methods.** |
| Study size | 10 | Explain how the study size was arrived at **This is in the first paragraph of the Results section.** |
| Quantitative variables | 11 | Explain how quantitative variables were handled in the analyses. If applicable, describe which groupings were chosen and why **This is in Methods under the Predictors of Kidney Function Recovery, first paragraph.** |
| Statistical methods | 12 | (*a*) Describe all statistical methods, including those used to control for confounding **This is in the Methods section, 2^nd^ and 3^rd^ paragraph under Predictors of Recovery of Kidney Function** |
| (*b*) Describe any methods used to examine subgroups and interactions **This is in the last paragraph under Predictors of Recovery of Kidney Function in Methods and also under the section *Subgroup analyses among diagnoses with high rates of recovery of kidney function*** |
| (*c*) Explain how missing data were addressed **This can be found in the second paragraph under Temporal Trends in Recovery of Kidney Function.** |
| (*d*) *Cohort study*—If applicable, explain how loss to follow-up was addressed ✔  **This is in the second paragraph under Predictors of Recovery of Kidney Function** |
| (*e*) Describe any sensitivity analyses **This is in the last paragraph under Definition of recovery and discontinuation of maintenance dialysis** |

Continued on next page

| Results | | |
| --- | --- | --- |
| Participants | 13* | (a) Report numbers of individuals at each stage of study—eg numbers potentially eligible, examined for eligibility, confirmed eligible, included in the study, completing follow-up, and analysed **This is in the first paragraph of the Results section.** |
|  |  | (b) Give reasons for non-participation at each stage **This is not a study that has any participation.** |
|  |  | (c) Consider use of a flow diagram **There were not many exclusions so we considered this but did not end up having a flow diagram.** |
| Descriptive data | 14* | (a) Give characteristics of study participants (eg demographic, clinical, social) and information on exposures and potential confounders This is under the Result section *Predictors of recovery of kidney function in adults and children,* Paragraphs 1-3. |
|  |  | (b) Indicate number of participants with missing data for each variable of interest. **This is provided in the legends to Table 2A and 2B.** |
|  |  | (c) *Cohort study*—Summarise follow-up time (eg, average and total amount) **Follow up time is in the first paragraph of the results.** |
| Outcome data | 15* | *Cohort study*—Report numbers of outcome events or summary measures over time. **This is in the first three sections of the results section.** |
|  |  | *Case-control study—*Report numbers in each exposure category, or summary measures of exposure**Not applicable** |
|  |  | *Cross-sectional study—*Report numbers of outcome events or summary measures **Not applicable.** |
| Main results | 16 | (*a*) Give unadjusted estimates and, if applicable, confounder-adjusted estimates and their precision (eg, 95% confidence interval). Make clear which confounders were adjusted for and why they were included **This is shown in Table 2A and 2B.** |
|  |  | (*b*) Report category boundaries when continuous variables were categorized. **This is shown in Table 2A and 2B and throughout all of the results section.** |
|  |  | (*c*) If relevant, consider translating estimates of relative risk into absolute risk for a meaningful time period. **This is not applicable.** |
| Other analyses | 17 | Report other analyses done—eg analyses of subgroups and interactions, and sensitivity analyses **These are reported in the Subgroup analyses among diagnoses section of Results.** |
| Discussion | | |
| Key results | 18 | Summarise key results with reference to study objectives **This is** **in the first paragraph of the discussion.** |
| Limitations | 19 | Discuss limitations of the study, taking into account sources of potential bias or imprecision. Discuss both direction and magnitude of any potential bias**This is in the second to last paragraph of the discussion.** |
| Interpretation | 20 | Give a cautious overall interpretation of results considering objectives, limitations, multiplicity of analyses, results from similar studies, and other relevant evidence **This is in the concluding paragraph.** |
| Generalisability | 21 | Discuss the generalisability (external validity) of the study results **This is in the second to last paragraph of the discussion.** |
| Other information | | |
| Funding | 22 | Give the source of funding and the role of the funders for the present study and, if applicable, for the original study on which the present article is based. **This is in the acknowledgements section.** |

*Give information separately for cases and controls in case-control studies and, if applicable, for exposed and unexposed groups in cohort and cross-sectional studies.

**Note:** An Explanation and Elaboration article discusses each checklist item and gives methodological background and published examples of transparent reporting. The STROBE checklist is best used in conjunction with this article (freely available on the Web sites of PLoS Medicine at http://www.plosmedicine.org/, Annals of Internal Medicine at http://www.annals.org/, and Epidemiology at http://www.epidem.com/). Information on the STROBE Initiative is available at www.strobe-statement.org.
