# Supplementary material for: Recovery of kidney function after dialysis initiation in children and adults in the US: A retrospective study of United States Renal Data System data
Source: PLoS Med. 2021 Feb 19;18(2):e1003546. doi: 10.1371/journal.pmed.1003546 (PMC7935284; doi:10.1371/journal.pmed.1003546)
Supplement: S1 Table — ESKD, end-stage kidney disease. (DOCX) [file pmed.1003546.s002.docx]

**Supplemental Table 1.** Adjusted* Fine and Gray models for time to recovery from maintenance dialysis at any point during follow-up after ESKD onset.

| **Characteristics** | **Multivariable model** | |
| --- | --- | --- |
|  | Sub-HR (95% CI) | P-value |
| Age category (years)  0-<5  5-<13  13-<18  18-30  30-65  65+ | 1.00 (0.73-1.37)  0.66 (0.47-0.92)  0.68 (0.60-0.77)  Reference  0.94 (0.74-1.21)  0.72 (0.56-0.92) | 0.99  0.01  <0.001  0.64  0.009 |
| Female | 1.00 (0.98-1.01) | 0.56 |
| Race  NHW  Black  Hispanic  Asian  Other | Reference  0.52 (0.51-0.53)  0.65 (0.64-0.67)  0.53 (0.50-0.55)  0.54 (0.50-0.57) | <0.001  <0.001  <0.001  <0.001 |
| Primary cause of kidney disease  ATN  AIN  Glomerulonephritis  Diabetes  Hypertension  Cystic/urologic/CAKUT  Etiology unknown  Other | Reference  1.19 (1.13-1.26)  0.20 (0.19-0.20)  0.10 (0.09-0.10)  0.15 (0.14-0.15)  0.10 (0.09-0.10)  0.24 (0.23-0.25)  0.27 (0.26-0.27) | <0.001  <0.001  <0.001  <0.001  <0.001  <0.001  <0.001 |
| Peritoneal dialysis (vs. hemodialysis) | 0.41 (0.39-0.42) | <0.001 |
| Calendar year  1996-2000  2001-2005  2006-2010  2011-2015 | Reference  1.35 (1.32-1.38)  1.86 (1.82-1.90)  1.71 (1.67-1.75) | <0.001  <0.001  <0.001 |
| Region of the US  West  Midwest  South  Northeast | Reference  0.96 (0.94-0.98)  1.09 (1.07-1.11)  0.72 (0.70-0.74) | <0.001  <0.001  <0.001 |
| Median income | 1.00 (1.00-1.00) | 0.001 |
| Insurance  None  Medicaid/Medicare  Private/Other | Reference  1.01 (0.92-1.10)  1.05 (0.96-1.14) | 0.87  0.33 |

*Adjusted additionally for CAD, malignancy, heart failure, diabetes, hypertension, PVD, stroke, drug use, tobacco use

Includes N=1933687 in fully adjusted analysis due to missing covariates

NHB = Non-Hispanic Black; NHW = Non-Hispanic White

CAKUT = congenital anomalies of the kidney and urinary tract; ATN = acute tubular necrosis

AIN = acute interstitial nephritis; ESKD = end-stage kidney disease
