# Supplementary material for: Recovery of kidney function after dialysis initiation in children and adults in the US: A retrospective study of United States Renal Data System data
Source: PLoS Med. 2021 Feb 19;18(2):e1003546. doi: 10.1371/journal.pmed.1003546 (PMC7935284; doi:10.1371/journal.pmed.1003546)
Supplement: S2 Table — ESKD, end-stage kidney disease. (DOCX) [file pmed.1003546.s003.docx]

**Supplemental Table 2.** Adjusted* Fine and Gray models for time to recovery from maintenance dialysis at any point during follow-up after ESKD onset with a focus on factors that differed between children and adults.

| **Multivariable models** | **Adults** | | **Children** | |
| --- | --- | --- | --- | --- |
|  | Sub-HR (95% CI) | P-value | Sub-HR (95% CI) | P-value |
| Age category (years)  0-<5  5-<13  13-<18  18-30  30-65  65+ | --  --  --  Reference  0.94 (0.91-0.98)  0.72 (0.69-0.74) | 0.001  <0.001 | 1.87 (1.50-2.34)  1.04 (0.84-1.28)  Reference  --  --  -- | <0.001  0.71 |
| Female | 0.99 (0.98-1.01) | 0.29 | 1.50 (1.28-1.77) | <0.001 |
| Race  NHW  Black  Hispanic  Asian  Other | Reference  0.52 (0.51-0.53)  0.66 (0.64-0.67)  0.53 (0.50-0.55)  0.53 (0.50-0.56) | <0.001  <0.001  <0.001  <0.001 | Reference  0.90 (0.72-1.12)  0.71 (0.57-0.90)  0.83 (0.51-1.35)  1.21 (0.75-1.95) | 0.33  0.004  0.45  0.43 |
| Primary cause of kidney disease  Glomerulonephritis  ATN  AIN  Diabetes  Hypertension  Cystic/urologic/CAKUT  Etiology unknown  Other | 0.19 (0.19-0.20)  Reference  1.19 (1.13-1.26)  0.10 (0.09-0.10)  0.15 (0.14-0.15)  0.10 (0.09-0.10)  0.24 (0.23-0.25)  0.27 (0.26-0.28) | <0.001  <0.001  <0.001  <0.001  <0.001  <0.001  <0.001 | 0.45 (0.32-0.63)  Reference  1.41 (0.29-6.81)  0.26 (0.08-0.78)  0.26 (0.14-0.47)  0.12 (0.07-0.20)  0.18 (0.10-0.30)  0.22 (0.16-0.31) | <0.001  0.67  0.02  <0.001  <0.001  <0.001  <0.001 |
| Peritoneal dialysis (vs. hemodialysis) | 0.40 (0.39-0.42) | <0.001 | 0.65 (0.55-0.77) | <0.001 |
| Calendar year  1996-2000  2001-2005  2006-2010  2011-2015 | Reference  1.35 (1.32-1.38)  1.86 (1.83-1.90)  1.72 (1.68-1.75) | <0.001  <0.001 | Reference  1.41 (1.11-1.79)  1.49 (1.18-1.88)  1.06 (0.83-1.37) | 0.005  0.001  0.63 |

*Adjusted additionally for median income, region, insurance type, and comorbidities (CAD, malignancy, heart failure, diabetes, hypertension, PVD, stroke, drug use, tobacco use) and accounting for the competing risk of death.

Includes N=1920951 adults and N=12736 children in fully adjusted analysis due to missing covariates

CAKUT = congenital anomalies of the kidney and urinary tract

NHB = Non-Hispanic Black; NHW = Non-Hispanic White

ATN = acute tubular necrosis

AIN = acute interstitial nephritis; ESKD = end-stage kidney disease
