# Supplementary figures and images for: Recovery of kidney function after dialysis initiation in children and adults in the US: A retrospective study of United States Renal Data System data
Source: PLoS Med. 2021 Feb 19;18(2):e1003546. doi: 10.1371/journal.pmed.1003546 (PMC7935284; doi:10.1371/journal.pmed.1003546)

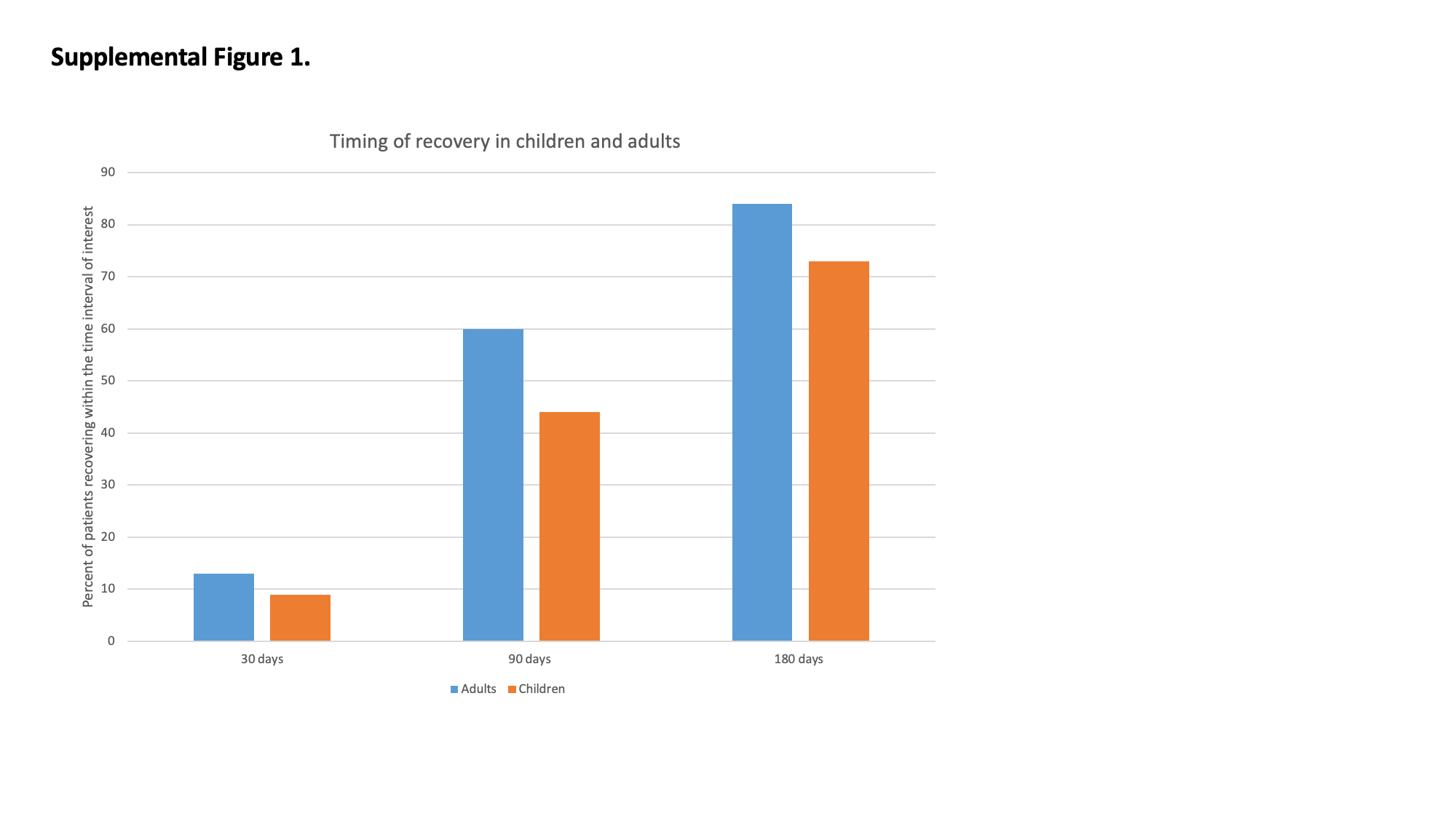

Supplement: S1 Fig — (TIF) [file pmed.1003546.s004.tif]
